# Epidemiological and anatomopathological profile of colorectal cancer: A cross-sectional study

**DOI:** 10.4102/jphia.v16i1.856

**Published:** 2025-03-21

**Authors:** Lahoucine Amsdar, Jamal Tikouk, Mohamed Amine Baba, Hafid Arzoug, Jaouad Elkhalladi, Salima Zerouali, Kenza Oqbani, Ghizlane Rais, Mehdi Soufi

**Affiliations:** 1 Laboratory of Biotechnology and Medicine, Faculty of Medicine and Pharmacy, Ibn Zohr University, Agadir, Morocco; 2 Applied Modeling in Economics and Management Laboratory, Faculty of Legal, Economic and Social Sciences Ain Sebaa, University of Hassan II, Casablanca, Morocco; 3 High Institute of Nursing Professions and Technical Health, Agadir, Morocco; 4 Research Laboratory in Endocrinology Gastroenterology Neuroscience Ethics, Faculty of Medicine and Pharmacy, Ibn Zohr University, Agadir, Morocco; 5 Oral Biology and Biotechnology Laboratory, Faculty of Medicine and Pharmacy, Mohammed V University, Rabat, Morocco; 6 Disciplinary Research Laboratory for Innovation in Teaching and Human Capital, Faculty of Educational Sciences, Mohammed V University, Rabat, Morocco; 7 Department of Pathology, Souss Massa University Hospital, Faculty of Medicine and Pharmacy, Ibn Zohr University, Agadir, Morocco; 8 Department of Medical Oncology, Faculty of Medicine and Pharmacy, Ibn Zohr University, Agadir, Morocco; 9 Department of Digestive and Visceral Surgery, University Hospital, Agadir, Morocco

**Keywords:** colorectal cancer, epidemiological profile, anatomopathological profile, Souss Massa, Morocco

## Abstract

**Background:**

Colorectal cancer (CRC) remains one of the leading causes of cancer-related deaths globally, with incidence and mortality rates exhibiting geographical disparities.

**Aim:**

This study aims to outline the pathological profile of CRC.

**Setting:**

The study was conducted in the anatomopathological laboratories of the Souss Massa region (SMR) in Morocco.

**Methods:**

The study examined the epidemiological and anatomopathological profile of CRC among patients diagnosed. We reviewed 238 anatomopathological results during the study period. Fisher’s exact test and analysis of variance were performed using Statistical Package for Social Sciences (SPSS) version 20.

**Results:**

Rectum and sigmoid colon were the most common sites for CRC (76.9%), with adenocarcinomas emerging as the predominant histological variant (93.3%). Most tumours were moderately differentiated (96.6%), with many (83.1%) in advanced stages (T3, T4). The presence of vascular embolism in 31.9% of patients indicates aggressive disease progression. Additionally, the study discerned a slight male dominance (52.9%) in the prevalence of CRC and an average age of 59 among patients. Notably, sex showed a significant association with the manifestation of CRC across various organs (*p* = 0.028), as did histological types across different organs (*p* = 0.010). Age-related analysis found older patients (over 50 years) with advanced-stage CRC more frequently.

**Conclusion:**

The histopathological features of these tumours are associated with an alarming delay in diagnosis and a significant presence of vascular embolism in patients.

**Contribution:**

Delay in diagnosis of CRC is significant in the SMR. There is an urgent need to strengthen screening strategies and examine social determinants of health for earlier diagnosis.

## Introduction

Globally, colorectal cancer (CRC) is witnessing a rapid rise in both diagnoses and fatalities, with 1.93 million individuals diagnosed and 0.94m deaths in 2020, making up 10% of all cancer cases and 9.4% of cancer deaths worldwide.^1^ It is the third highest cause of cancer mortality globally, with deaths numbering 515 637 in men and 419 536 in women for the year.^1^ Presently, the 5-year prevalence of CRC exceeds 5.25m cases, second only to breast cancer. Advances in understanding CRC’s mechanisms and improvements in various treatments, including endoscopic and surgical interventions, targeted and radiation therapy, along with chemotherapy and immunotherapy, have improved the 3-year survival rate for those with advanced stages of the disease.^2^ The screening of CRC has been revealed as the most important aspect in smoothing the incidence and mortality of this disease.^3^ Indeed, the largest reductions in CRC mortality have been observed in countries where screening programmes have existed for a long time.^4^

In Morocco, it ranks as the second leading cause of digestive cancer, following gastric cancer. Despite its lower incidence compared to Western countries, with a rate of 2.5 to 3.3 per 100 000 people, it matches the rates found in other Maghreb countries. Notably, in 27% of the cases within these regions, the disease affects younger individuals.^5^

A study conducted at the University Hospital Center (UHC) of Fez on 5532 new cancer cases across all locations found that the digestive system is the most common site, with 1120 cases, accounting for 20.25% of all cases. Colorectal cancers are particularly notable for their high frequency, comprising 464 cases (41.43%).^6^

In terms of histopathology, the left colon was more frequently affected, accounting for 74.28% of cases. Histopathological examination revealed 27 cases of adenocarcinomas not otherwise specified (NOS), including six well-differentiated, 20 moderately differentiated and one poorly differentiated. Additionally, there were eight cases of mucinous adenocarcinomas.^7^ Also, the initiation and development of tumorigenesis from normal colonic mucosa to a fully developed carcinoma and metastasis are typically linked with specific histopathological and morphological characteristics. Adenocarcinoma is the most prevalent type of CRC tumour type.^8^

Similarly, histological aspects such as tumour budding, perineural invasion and lymph node involvement, along with molecular markers such as Kirsten rat sarcoma virus (KRAS), B-Raf proto-oncogene (BRAF), microsatellite instability (MSI) and CDX2 can help to establish a prognosis and optimise treatment.^9^

Recently, it is worth mentioning that several studies have focussed on describing the epidemiological and anatomopathological profile of patients with CRC in different regions in Morocco.^10,11,12,13^ Nevertheless, in the Southern region including Souss Massa, there is a paucity of data on CRCs, which can best describe our epidemiological profile and guide us in tailored and effective preventive, diagnostic and management strategies. Therefore, this study was instituted in order to determine the epidemiological and anatomopathological characteristics of CRCs in this region. By achieving this objective, this research will contribute to filling a notable gap in the epidemiological scientific literature in Morocco, which could empower policymakers to design and implement more tailored and effective preventing health interventions in order to reduce the incidence of CRC in Morocco.^14,15^

The ultimate objective of the present study is to describe the epidemiological and pathological aspects of CRC in the Souss Massa Region (SMR) in order to build a usable database that will be a source of information for the orientation of the prevention and therapeutic strategies put in place.

## Research methods and design

### Study site

**FIGURE 1:**
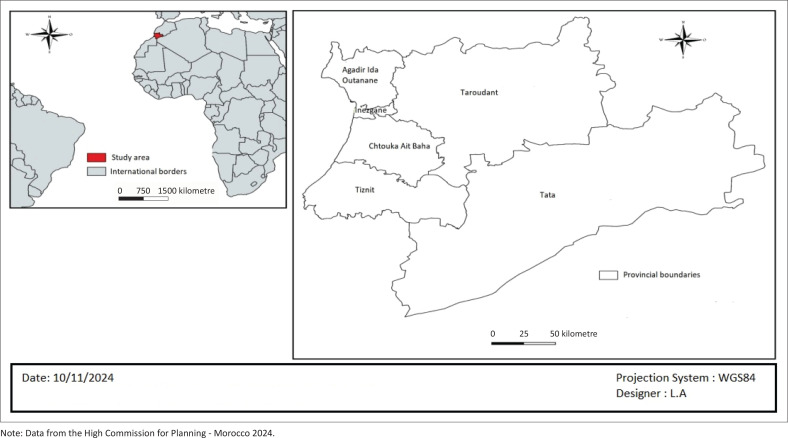
Thematic map of the Souss Massa region - Kingdom of Morocco.

The study was conducted in the SMR, a geographically diverse area with a population of approximately 2.7m. The region’s unique environmental and socioeconomic factors may influence CRC incidence and management^16^ (Figure 1).

### Type of study

This is a descriptive retrospective analytical study conducted in the anatomopathology laboratories of the SMR involving 238 patients diagnosed with CRC in SMR. The retrospective approach enabled the examination of the anatomopathological reports.

### Data sources

The data sources used in this study included anatomopathological reports. These sources provided a foundation for the research, enabling a detailed analysis of patient epidemiological and anatomopathological characteristics.

### Sampling

Patients were randomly selected using Microsoft Excel. The sample size was calculated using the Online Roasfot platform, adhering to a Type I error (α) of 5% and a statistical power of 80% (*β* = 0.20), with a margin of error set at 5%. Given a total population of 620 medical reports, the minimum required sample size is determined using the Stephen Thompson equation for finite populations:

$$n=\frac{n\;Z^{2}\;p(1-P)}{\left(N-1)E^{2}+Z^{2}p(1-P\right)}$$ [Eqn 1]

where *n* = required sample size; *N* = total population size (in this case, 620 medical reports); *Z* = *Z*-score for the desired confidence level (for α = 0.05, *Z* ≈ 1.96); *p* = estimated proportion (0.5 if unknown) and *E* = margin of error (expressed as a decimal, so 5% = 0.05).

### Data analysis

Data were cleaned and analysed using Statistical Package for Social Sciences (SPSS) version 20. Descriptive statistics were calculated for patient demographics and tumour characteristics. Fisher’s exact test and analysis of variance (ANOVA) were used to compare groups. Statistical significance was set at *p* < 0.05.

### Epidemiological and anatomopathological characteristics

The dataset included patient demographics (age, gender, location and the area of residence), CRC characteristics (organ, location, type of sampling, histological type, differentiation, stage and MSI status) and disease progression indicators (vascular embolism and perineural invasion).

### Inclusion and exclusion criteria

The study on CRC in the SMR specified inclusion criteria that limited participants to patients with a histologically confirmed diagnosis of CRC from anatomopathology laboratories affiliated between 01 January 2021 and 31 December 2023. To ensure the focus remained on malignant cases of CRC, several exclusion criteria were rigorously applied: patients diagnosed with other digestive cancers were omitted to maintain diagnostic specificity; patients with adenomatous polyps associated with CRC were also excluded. Consequently, 22 patients were precisely excluded based on these criteria: non-CRC-related digestive cancers (1.15%), adenomatous polyps (5.38%) and incomplete pathological reports (1.92%). Following these exclusions, the study population was reduced to 238 participants from an initial 260, achieving a participation rate of 91.53%.

### Ethical considerations

Ethical clearance to conduct this study was obtained from the Mohamed V University of Rabat (reference no.: 18/24). Oral informed consent was obtained from patients. Data were anonymised and permission was granted to collect data from all pathology laboratories in the region.

## Results

### Descriptive analysis

The distribution by gender shows a slightly higher percentage of men (52.9%) than women (47.1%), indicating a relatively balanced gender ratio within the studied sample. The average age is 59 years, suggesting that the population is primarily composed of older patients. This balanced gender distribution is typical in many studies of CRC, reflecting the broad impact of the disease across both sexes. The predominance of older adults in the sample aligns with global epidemiological trends that show CRC is more commonly diagnosed in individuals over 50 years of age (Table 1).

**TABLE 1:** Epidemiological characteristics of patients with colorectal cancer (*N* = 238).

Variable	*n*	%	Mean ± s.d.
**Gender**			
Male	126	52.9	-
Female	112	47.1	-
**Age (years)**	-	-	59 ± 12.18
**Province**			
Agadir Ida Outanane	81	34.0	-
Inezgane Ait Melloul	37	15.5	-
Chtouka Ait Baha	11	4.6	-
Tiznit	15	6.3	-
Taroudannt	76	31.9	-
Tata	3	1.3	-
Others	15	6.3	-
**Residence area**			
Urban	152	63.9	-
Rural	86	36.1	-

s.d., standard deviation.

**FIGURE 2:**
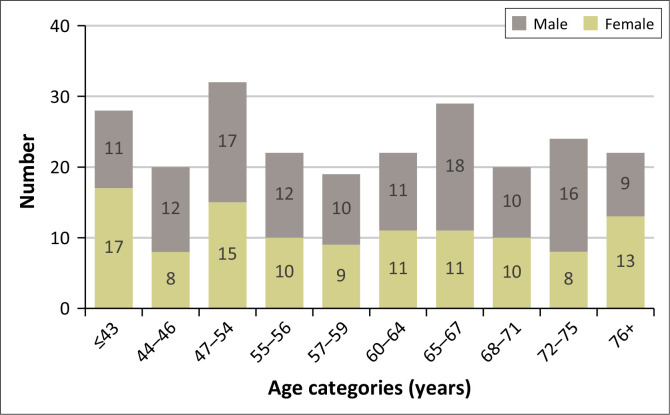
Distribution of patients’ age across gender.

The distribution of patients across age categories and gender reveals notable disparities, particularly between different age groups, except when the age is over 76 years and under 43 years or less (Figure 2).

In terms of provincial distribution, it is revealed that the population is spread across several provinces of SMR in Morocco, with Agadir Ida Outanane and Taroudant having the highest representations at 34.0% and 31.9%, respectively. As for the residence of patients, the data show a predominance of urban residence (63.9%) compared to rural residence (36.1%).

These findings shed light on the impact of demographic factors such as age, gender and place of residence on the distribution of CRC patients within the region. The higher incidence in urban areas may reflect differences in lifestyle factors, access to healthcare or environmental exposures compared to rural areas. Moreover, the significant representation from specific provinces could suggest areas where targeted public health interventions and increased screening and healthcare resources might be particularly beneficial.

### Anatomopathological profile

The provided statistics on the primary locations of CRC show a significant concentration in the sigmoid colon (37.0%) and rectum (39.9%), together accounting for over three-quarters of the cases. Lower percentages are observed in the ascending colon (6.7%), the cecum (4.6%) and the transverse colon (3.4%). The small intestine and descending colon each represent a smaller proportion of cases (3.4% and 2.5%, respectively). Notably, in cases where conditions extend across multiple locations, such as the descending colon and cecum (0.4%), the transverse colon, left colon, rectum (0.8%) and the ascending colon, middle rectum (0.4%).

The distribution of sample types in this sample reveals a significant reliance on biopsies, which account for 58.8% of the samples taken. Surgical resections constitute a substantial portion of the remaining samples, with a standard colectomy representing 16.8%. Hemicolectomy and segmental resection represent 5.9% and 10.9%, respectively. The lower percentages have been noted for right colectomy (1.3%), left colectomy (0.8%), right ileocecectomy (2.9%) and total colectomy (2.5%).

The most frequently reported macroscopic aspect is ‘ulcerated, circumferential, and stenosing budding’, representing 33.6% of the cases. The second most common aspect is ‘ulcerated and polypoid budding’, which accounts for 25.6% of the cases. Other aspects such as ‘ulcerated-budding’ alone (12.2%) and ‘ulcerated and circumferential budding’ (8.4%) also represent significant portions. Less common characteristics include ‘infiltrating’ (5.5%), ‘ulcerated and infiltrating’ (3.8%) and various combinations involving occlusive, mucoid and whitish features.

Adenocarcinomas (93.3%) represent the vast majority of cases. This prevalence shows that adenocarcinoma is the most common type of CRC. The other type of category (6.7) encompasses all types other than adenocarcinoma, which may include squamous cell carcinomas, neuroendocrine tumours and other rarer histological types. A small fraction of tumours (2.9%) is highly differentiated, meaning the cancer cells closely resemble normal cells in appearance and function.

The majority of tumours (96.6%) fall into this category, indicating a moderate resemblance to normal cells. Although these tumours may grow and spread at a moderate pace, the high prevalence of moderately differentiated cancers suggests a standard characteristic of CRC, influencing treatment planning and outcomes. Very few tumours (0.4%) are poorly differentiated, where cancer cells barely resemble their tissue of origin.

Early-stage cancers (T1 and T2), which indicate limited tumour invasion depth, constitute a small part of the cases, with only 0.4% in T1 and 12.7% in T2. A large majority of cases are diagnosed at more advanced stages, with 60.6% in T3 and 22.5% in T4. Only a small percentage of cases (3.8%) do not have specified stages.

A portion of the cases has a defined stability status (12.6%), with the majority not specified. Vascular embolism, indicating the presence of cancer cells (31.9%) in the vessels, is noted in a significant number of cases, which may be a sign of aggressive disease and metastatic potential.

The presence of vascular embolism in nearly a third of the cases underscores the importance of thorough vascular examination in CRC. A small proportion of cases (5.0%) presents invasion of the peripheral nerve sheath, a marker of aggressive disease and potential for pain or other neurological symptoms.

The balanced rates of presence and absence among specified cases indicate that this feature is not predominant, but it is still significant for those affected (Table 2).

**TABLE 2:** Anatomopathological characteristics of patients with colorectal cancer.

Variable	*n*	%
**Primary location**		
Ascending colon	6	2.50
Descending colon	88	37.00
Sigmoid colon	8	3.40
Transverse colon	8	3.40
Small intestine	11	4.60
Caecum	1	0.40
Descending colon and caecum	2	0.80
Transverse colon, left colon and rectum	95	39.90
Rectum	2	0.80
Rectosigmoid	1	0.40
Ascending colon and mid rectum	6	2.50
**Type of sampling**		
Biopsies	140	58.80
Right colectomy	3	1.30
Left colectomy	2	0.80
Colectomy	40	16.80
Hemicolectomy	14	5.90
Right ileo-colectomy	7	2.90
Segmental colectomy	0	0.00
Segmental resection	26	10.90
Total colectomy	6	2.50
**Histological type**		
Adenocarcinoma	222	93.30
Others	16	6.70
**Degree of differentiation**		
Well differentiated	7	2.90
Moderately differentiated	230	96.60
Poorly differentiated	1	0.40
**TNM staging**		
T1	1	0.40
T2	30	12.70
T3	143	60.60
T4	53	22.50
Not specified	9	3.80
**MSI status**		
Stable	30	12.60
Unstable	8	3.40
Not specified	200	84.00
**Vascular emboli**		
No	31	13.00
Yes	76	31.90
Not specified	131	55.00
**Perineural invasion**		
No	13	5.50
Yes	12	5.00
Not specified	213	89.50

Note: T1, T2, T3, T4 stages of cancer (derived from the 2017 edition of International Union Against Cancer classification).

MSI, microsatellite instability; TNM, tumour-node-metastasis.

### Bivariate analysis

Table 2 presents the association between different tumour sites categorised by gender. According to Fisher’s exact test performed, there is a significant association between gender and the occurrence of cases across the tumour site examined with a *p*-value of 0.28 suggesting a statistically significant association (Table 3).

**TABLE 3:** Association between gender and tumour site.

Tumour site	Gender		Association test
	Male	Female	
Colon	66	77	6.705*
Rectum	57	33	2**
Rectosigmoid junction	3	2	0.028***

**Total**	**126**	**112**	**-**

* , Value of test exact of Fisher;

** , number of degrees of freedom;

*** , *p* < 0.05.

Fisher’s exact test revealed a *p*-value of 0.010, indicating that the distribution of histological types varies significantly from one tumour site to another. Specifically, the majority of adenocarcinoma cases were observed in the colon, with 129 cases out of a total of 143 cases in this tumour site. The rectum and the rectosigmoid junction had fewer cases of adenocarcinoma, with 89 out of 90 cases and 4 out of 5 cases, respectively. Other histological types were also present although in smaller numbers (Table 4).

**TABLE 4:** The association histology type and tumour site.

Tumour site	Histology type		Total	Association test
	Adenocarcinoma	Other		
Colon	129	14	143	9.520*
Rectum	89	1	90	2**
Rectosigmoid junction	4	1	5	0.010***

**Total**	**222**	**16**	**238**	**-**

* , Value of test exact of Fisher;

** , number of degrees of freedom;

*** , *p* < 0.05.

Regarding the association between CRC and the stage of the disease, an ANOVA was conducted to examine the differences in average age across four cancer stages labelled T1, T2, T3 and T4. The descriptive statistics revealed that patients in stage T1 had an observation with an average age of 57 years, while those in stage T2 included 30 patients with an average age of 51.03 years (standard deviation [s.d.] = 10.829). Stage T3 included 143 patients with an average age of 58.89 years (s.d. = 12.167) and stage T4 included 53 patients with an average age of 65.58 years (s.d. = 10.018). The ANOVA yielded a statistically significant result, F(3.232) = 10.459, *p* < 0.001, showing differences in average age within the sample (Table 5).

**TABLE 5:** Comparison between colorectal cancer stage and patient age.

Stage	*n*	Mean	s.d.	ANOVA test
T1	1	57.00	-	10.459*
T2	30	51.03	10.829	0.000**
T3	143	58.89	12.167	-
T4	53	65.58	10.018	-

**Total**	**236**	**59.31**	**12.203**	-

Note: T1, T2, T3, T4, stages of cancer (derived from the 2017 edition of UICC classification).

ANOVA, analysis of variance; s.d., standard deviation.

* , Value of test exact of Fisher;

** , *p* < 0.05.

## Discussion

### Discussion of main results

Colorectal cancer represents a serious public health issue in terms of diagnosis, treatment and prevention in Morocco. This research aims to describe the epidemiological and anatomopathological profile of patients with CRC in SMR Morocco. The main results of this research have shown that the average age of patients diagnosed with CRC is about 59 years, with a standard deviation of 12.18 years. There is a slight difference between men (59.53 years) and women (57 years) diagnosed with CRC in the SMR.^17^ According to the Moroccan literature on the subject, the average age of patients with CRC is around 56 years, with a notable distribution between the sexes: 57.9% men and 42.1% women, indicating a slight male predominance in the incidence of CRC. However, these results are consistent with those of other studies, which note that the peak frequency of CRC occurs between 50 and 59 years in women, while in men, it occurs between 60 and 69 years.^18,19,20^

Additionally, CRC affects a significant number of younger patients, with studies revealing a considerable proportion of patients under 50 years old, highlighting the impact of the disease on both older and younger populations. The pathological characteristics, such as the prevalence of mucinous and signet ring cell types in younger patients. Similarly, another study conducted by Haimer et al. (2019) showed variable incidence rates across regions of Morocco. The disease affects both men and women, with a notable prevalence in patients aged 40 to 59 years. The majority of cases are presented at an advanced stage because of late diagnosis and limited screening practices. Regarding socioeconomic status (SES) and educational levels, the findings revealed a significant impact on the incidence of CRC and the stages at which it is diagnosed. Patients from lower SES groups and those with limited educational levels are at a higher risk of developing CRC and are often diagnosed at more advanced stages, underlining the importance of targeted awareness and screening programmes.^18,21^ In the same context, the challenges of screening and management have shown that the coronavirus disease 2019 (COVID-19) pandemic has had a significant impact on the screening and management of CRC, leading to delays in diagnosis and treatment.^14,22^

Recent research emphasises the key role of understanding the distribution and characteristics of CRC across different parts of the colon and rectum, which impacts diagnosis, treatment planning and patient prognosis.^23^ A similar study shows the predominance of CRC in the left colon and rectum compared to the right colon in an Indian population, with moderately differentiated adenocarcinoma being the most common histological type.^7^ This regional prevalence aligns with findings from another study in the United States, which reported distinct metastatic profiles for colon and rectal cancers, influencing treatment and monitoring strategies.^24^ For instance, colon cancer exhibited a higher rate of liver metastases, while rectal cancer was more likely to metastasise to the lungs and bones. Similarly, another research conducted a population-based study in Sweden, further elucidating the distinct metastatic patterns between colon and rectal cancers, which could significantly assist in patient monitoring and understanding the mechanisms of metastasis.^25,26^

### Study limitations

The study was limited to the analysis of anatomopathological data, omitting a detailed exploration of socioeconomic factors that could influence the stage at which cancer is diagnosed. This omission overlooks the potential insights these variables could provide for understanding cancer prognosis.

### Research perspective

While this study offers insights into CRC in the SMR, it has limitations. Further research is needed to comprehensively understand CRC in Morocco. This includes nationwide studies, examining social determinants of cancer screening and longitudinal studies to track CRC incidence.

## Conclusion

Colorectal cancer is the third most common cancer worldwide, affecting both men and women. Incidence and mortality rates vary significantly from one country to another. These variations can be attributed to several factors, including differences in risk factors, access to healthcare, the quality of healthcare systems and screening programmes. This research aimed to outline the epidemiological and anatomopathological profile of patients with CRC at the anatomopathology laboratories in the SMR. The results highlight the characteristics of CRC from an epidemiological and anatomopathological viewpoint. It was revealed that the sigmoid colon (37.0%) and the rectum (39.9%) are the most common sites for these conditions, with the majority of samples (58.8%) being obtained through biopsy. The most frequently identified tumour characteristic was ‘ulcerated-budding, circumferential, and stenosing’ (33.6%). In terms of histology, adenocarcinomas were predominant (93.3%), with a significant proportion of tumours (96.6%) showing moderate differentiation. The study also highlighted that late diagnosis is common, with advanced stages (T3 and T4) accounting for 83.1% of diagnoses. Moreover, the presence of vascular embolism in 31.9% of cases indicates a considerable probability of aggressive disease progression.^27,28^

## References

[1] Morgan E, Arnold M, Gini A, et al. Global burden of colorectal cancer in 2020 and 2040: Incidence and mortality estimates from GLOBOCAN. Gut. 2023;72(2): 338–344. 10.1136/gutjnl-2022-327736 36604116

[2] Dekker E, Tanis PJ, Vleugels JLA, Kasi PM, Wallace MB. Colorectal cancer. Lancet. 2019;394(10207):1467–1480. 10.1016/S0140-6736(19)32319-0 31631858

[3] Schreuders EH, Ruco A, Rabeneck L, et al. Colorectal cancer screening: A global overview of existing programmes. Gut. 2015;64(10):1637–1649. 10.1136/gutjnl-2014-309086 26041752

[4] Cardoso R, Guo F, Heisser T, et al. Colorectal cancer incidence, mortality, and stage distribution in European countries in the colorectal cancer screening era: An international population-based study. Lancet Oncol. 2021;22(7):1002–1013. 10.1016/S1470-2045(21)00199-6 34048685

[5] Belhamidi MS, Sinaa M, Kaoukabi A, et al. Profil épidémiologique et anatomopathologique du cancer colorectal: à propos de 36 cas. Pan Afr Med J. 2018;30:159. 10.11604/pamj.2018.30.159.15061 30455788 PMC6235491

[6] Chbani L, Hafid I, Berraho M, Mesbahi O, Nejjari C, Amarti A. Aspects épidémiologiques et anatomopathologiques des cancers dans la région de Fès-Boulemane (Maroc). Eastern Mediterranean Health J. 1995:263–270. 23879078

[7] Kalita NP, Khakhlari S, Das S, Boro G. Histopathological evaluation of right and left sided colorectal cancer: A cross-sectional study from a Tertiary Care Hospital, Assam, India. J Clin Diagn Res. 2022;16(7):EC32–EC35. 10.7860/JCDR/2022/55775.16656

[8] Treanor D, Quirke P. Pathology of colorectal cancer. Clin Oncol. 2007;19(10): 769–776. 10.1016/j.clon.2007.08.012 17950585

[9] Chen K, Collins G, Wang H, Toh JWT. Pathological features and prognostication in colorectal cancer. Curr Oncol. 2021;28(6):5356–5383. 10.3390/curroncol28060447 34940086 PMC8700531

[10] Belhamidi MS, Sinaa M, Kaoukabi A, et al. [Epidemiological and pathological profile of colorectal cancer: About 36 cases]. Pan Afr Med J. 2018;30:159. 10.11604/pamj.2018.30.159.15061 30455788 PMC6235491

[11] Imad FE, Drissi H, Tawfiq N, et al. [Epidemiological, nutritional and anatomopathological features of patients with colorectal cancer in the greater Casablanca region]. Pan Afr Med J. 2019;32:56. 10.11604/pamj.2019.32.56.10548 31223348 PMC6561128

[12] Benmoussa A, Zamiati S, Badre W, et al. Colorectal cancer: Comparison of clinicopathologic features between Moroccans patients less than 50 years old and older. Pathol Biol. 2013;61(3):117–119. 10.1016/j.patbio.2012.01.003 22361163

[13] Haimer A, Belamalem S, Habib F, Mokhtari A, Soulaymani A, Hami H. Colorectal cancer in Morocco: Results of a retrospective study. Biosci Biotechnol Res Asia. 2019;16(1):79–83. 10.13005/bbra/2723

[14] Ouaamr A, Fechtali T. The influence of covid 19 on screening and management of colorectal cancers, case study of the agadir oncology centre. Int J Res Ethics. 2022;5(1):136. 10.51766/ijre.v5i1.136

[15] Tazi MA, Er-Raki A, Benjaafar N. Cancer incidence in Rabat, Morocco. Ecancermedicalscience. 2013;7:338. 23940493 10.3332/ecancer.2013.338 PMC3737118

[16] High commissariat of p. Monography of The region Souss Massa [homepage on the Internet]. [cited n.d.]. Available from: https://www.hcp.ma/Recensement-population-RGPH-2014_a2941.html

[17] El Kinany K, Huybrechts I, Kampman E, et al. Concordance with the World Cancer Research Fund/American Institute for Cancer Research recommendations for cancer prevention and colorectal cancer risk in Morocco: A large, population-based case–control study. Int J Cancer. 2019;145(7):1829–1837. 10.1002/ijc.32263 30861106

[18] Haimer A, Belamalem S, Habib F, Soulaymani A, Mokhtari A, Hami H. Colorectal cancer in Morocco: Results of a retrospective study. Biosci Biotechnol Res Asia. 2019;16(1):79–83. 10.13005/bbra/2723

[19] Tebibel S, Zouaghi Y, Atallah S, Mechati C, Messaoudi S, Kabbouche S. Colorectal cancer: Epidemiological study, clinical, pathological an immunohistochemical Examination in patients of Eastern Algeria. Int J Pharm Sci Rev. 2014;26(2):13–18.

[20] Imad FE, Drissi H, Tawfiq N, Bendahhou K, Benider A, Radallah D. Facteurs de risque alimentaires du cancer colorectal au Maroc: étude cas témoin. Pan Afr Med J. 2020;35:59. 10.11604/pamj.2020.35.59.18214 PMC7250211 32537063

[21] Leufkens AM, Van Duijnhoven FJB, Boshuizen HC, et al. Educational level and risk of colorectal cancer in EPIC with specific reference to tumor location. Int J Cancer. 2012;130(3):622–630. 21412763 10.1002/ijc.26030

[22] Yong JH, Mainprize JG, Yaffe MJ, et al. The impact of episodic screening interruption: COVID-19 and population-based cancer screening in Canada. J Med Screen. 2021;28(2):100–107. 10.1177/0969141320974711 33241760 PMC7691762

[23] Holch JW, Ricard I, Stintzing S, Modest DP, Heinemann V. The relevance of primary tumour location in patients with metastatic colorectal cancer: A meta-analysis of first-line clinical trials. Eur J Cancer. 2017;70:87–98. 10.1016/j.ejca.2016.10.007 27907852

[24] Liška V, Emingr M, Skála M, et al. Liver metastases from colon and rectal cancer in terms of differences in their clinical parameters. Rozhledy v Chirurgii: Mesicnik Ceskoslovenske Chirurgicke Spolecnosti. 2016;95(2):69–77. 27008168

[25] Qiu M, Hu J, Yang D, Cosgrove DP, Xu R. Pattern of distant metastases in colorectal cancer: A SEER based study. Oncotarget. 2015;6(36):38658–38666. 10.18632/oncotarget.6130 26484417 PMC4770727

[26] Riihimäki M, Hemminki A, Sundquist J, Hemminki K. Patterns of metastasis in colon and rectal cancer. Sci Rep. 2016;6(1):29765. 10.1038/srep29765 27416752 PMC4945942

[27] Ferlay J, Steliarova-Foucher E, Lortet-Tieulent J, et al. Cancer incidence and mortality patterns in Europe: Estimates for 40 countries in 2012. Eur J Cancer. 2013;49(6):1374–1403. 10.1016/j.ejca.2012.12.027 23485231

[28] Arnold M, Sierra MS, Laversanne M, Soerjomataram I, Jemal A, Bray F. Global patterns and trends in colorectal cancer incidence and mortality. Gut. 2017;66(4):683–691. 10.1136/gutjnl-2015-310912 26818619

